# Laboratory Methods for Detection of Infectious Agents and Serological Response in Humans With Tick-Borne Infections: A Systematic Review of Evaluations Based on Clinical Patient Samples

**DOI:** 10.3389/fpubh.2021.580102

**Published:** 2021-09-20

**Authors:** Anna J. Henningsson, Audun Aase, Herjan Bavelaar, Signe Flottorp, Pia Forsberg, Ingvild Kirkehei, Matilda Lövmar, Kenneth Nilsson, Dag Nyman, Katharina Ornstein, Johanna Sjöwall, Barbro H. Skogman, Ivar Tjernberg, Ingeborg Aaberge

**Affiliations:** ^1^Division of Clinical Microbiology, Laboratory Medicine, Region Jönköping County, Jönköping, Sweden; ^2^Division of Clinical Microbiology, Region Östergötland, Linköping, Sweden; ^3^Division of Inflammation and Infection, Department of Biomedical and Clinical Sciences, Linköping University, Linköping, Sweden; ^4^Division of Infection Control and Environmental Health, Norwegian Institute of Public Health, Oslo, Norway; ^5^Division of Health Services, Norwegian Institute of Public Health, Oslo, Norway; ^6^Division of Infectious Medicine, Department of Biomedical and Clinical Sciences, Linköping University, Linköping, Sweden; ^7^Norwegian College of Policing, Oslo, Norway; ^8^Department of Medical Sciences, Section of Clinical Microbiology, Uppsala University, Uppsala, Sweden; ^9^The Åland Group for Borrelia Research, Mariehamn, Finland; ^10^Ystad Hospital, Skåne University Health Care, Kristianstad, Sweden; ^11^Department of Infectious Diseases, Region Östergötland, Norrköping, Sweden; ^12^Department of Pediatrics and Center for Clinical Research, Dalarna-Uppsala University, Falun, Sweden; ^13^Faculty of Medical and Health Sciences, Örebro University, Örebro, Sweden; ^14^Department of Clinical Chemistry and Transfusion Medicine, Region Kalmar County, Kalmar, Sweden

**Keywords:** systematic review, tick-borne infections, co-infections, human, laboratory, diagnostic, clinical evaluation

## Abstract

**Background:** For the most important and well-known infections spread by *Ixodes* ticks, Lyme borreliosis (LB) and tick-borne encephalitis (TBE), there are recommendations for diagnosis and management available from several health authorities and professional medical networks. However, other tick-borne microorganisms with potential to cause human disease are less known and clear recommendations on diagnosis and management are scarce. Therefore, we performed a systematic review of published studies and reviews focusing on evaluation of laboratory methods for clinical diagnosis of human tick-borne diseases (TBDs), other than acute LB and TBE. The specific aim was to evaluate the scientific support for laboratory diagnosis of human granulocytic anaplasmosis, rickettsiosis, neoehrlichiosis, babesiosis, hard tick relapsing fever, tularemia and bartonellosis, as well as tick-borne co-infections and persistent LB in spite of recommended standard antibiotic treatment.

**Methods:** We performed a systematic literature search in 11 databases for research published from 2007 through 2017, and categorized potentially relevant references according to the predefined infections and study design. An expert group assessed the relevance and eligibility and reviewed the articles according to the QUADAS (diagnostic studies) or AMSTAR (systematic reviews) protocols, respectively. Clinical evaluations of one or several diagnostic tests and systematic reviews were included. Case reports, non-human studies and articles published in other languages than English were excluded.

**Results:** A total of 48 studies fulfilled the inclusion criteria for evaluation. The majority of these studies were based on small sample sizes. There were no eligible studies for evaluation of tick-borne co-infections or for persistent LB after antibiotic treatment.

**Conclusions:** Our findings highlight the need for larger evaluations of laboratory tests using clinical samples from well-defined cases taken at different time-points during the course of the diseases. Since the diseases occur at a relatively low frequency, single-center cross-sectional studies are practically not feasible, but multi-center case control studies could be a way forward.

## Introduction

The European tick *Ixodes ricinus* is the vector of several potential human pathogens, of which *Borrelia burgdorferi* sensu lato (s.l.) and tick-borne encephalitis virus (TBEV) are the most important and well-known in human medicine. The diagnosis of the diseases they may cause, Lyme borreliosis (LB) and tick-borne encephalitis (TBE), is based on the patients' medical history and clinical signs and symptoms together with laboratory support, which mainly consists of serology, sometimes supplemented with molecular detection by PCR. Both for LB and for TBE, clinical case definitions and recommendations for management are available from several health authorities and professional medical networks [e.g., (1–5)], with the exception of diagnostic methods for detection of possible persisting *Borrelia* infection in patients with remaining symptoms after antibiotic treatment of LB. Other tick-borne microorganisms with potential to cause human disease are less known and clear recommendations on diagnosis and management are scarce. Potential human pathogens that have been found in *I. ricinus* in northern or central Europe are for example *Anaplasma phagocytophilum* (6–8), *Rickettsia* spp. (6, 9, 10), *Neoehrlichia mikurensis* (11–13), *Babesia* species (spp.) (14, 15), *Borrelia miyamotoi* (16–18), *Francisella tularensis* (19–21) and *Bartonella* spp. (22–24). Several of these have the potential to cause severe disease, especially in immunocompromised patients [*A. phagocytophilum*: (25, 26); *Babesia* spp.: (27–30); *B. miyamotoi*: (31, 32); *N. mikurensis*: (33); *Rickettsia* spp.: (34, 35)], while their medical importance in immunocompetent individuals is more uncertain. Reports on seropositivity in tick-exposed populations without a known history of disease [*A. phagocytophilum:* (7, 36); *Babesia* spp.: (37, 38); *B. miyamotoi:* (39); multiple tick-borne pathogens: (40); *Bartonella* spp.: (41); *N. mikurensis:* (42); *F. tularensis:* (43); *Rickettsia* spp.: (44, 45)] indicate that exposure to several of these microorganisms does not always entail symptoms, or perhaps only causes mild and self-limiting symptoms. On the other hand, signs and symptoms like fever, skin rash, neutropenia, leukopenia, elevated liver enzymes, lymphadenopathy and even CNS infection, have also been reported in immunocompetent patients [*A. phagocytophilum:* (26, 46); *Babesia* spp.: (47); *B. miyamotoi:* (48–50); *N. mikurensis:* (51, 52); *F. tularensis:* (53); *Rickettsia* spp.: (54)], and consequently, a certain under-diagnosis of these infections must be suspected. Co-infections with more than one tick-borne pathogen have been reported [e.g., (28, 55–57)], but are probably in most cases overlooked in clinical practice. Recommendations regarding clinical and laboratory investigation of possible tick-borne co-infections are scarce and general guidelines are lacking. The scarcity of well-established guidelines for diagnosis and management of several of the tick-borne diseases (TBDs) contribute to the existing medical controversies in this field.

In 2015, the Norwegian Directorate of Health initiated a Nordic consensus collaboration focusing on diagnosis and management of TBDs other than LB and TBE, led by the Norwegian National Advisory Unit on Tick-borne Diseases. The Nordic consensus network consisted of physicians and researchers from Norway, Sweden, Denmark and Finland, as well as representatives from patient organizations. As part of this work, the Norwegian Institute of Public Health was engaged to perform a systematic literature search on clinical studies evaluating laboratory methods for diagnosis of human TBDs other than LB and TBE, and a group of physicians from the Nordic countries, all with clinical and research experience of TBDs, were assigned the task of reviewing the relevant references. The review process was observed by representatives from the Public Health Agency of Sweden, the Swedish Medical Products Agency, and the National Board of Health and Welfare in Sweden.

The purpose of this present systematic review was to provide an overview of published research from 2007 through 2017 on the performance of laboratory tests evaluated on clinical samples (i.e., using authentic patient samples) for the diagnosis of human TBDs, other than untreated LB and TBE, including laboratory diagnosis of tick-borne co-infections and post-treatment persisting LB, with the objective to elucidate the following clinical questions:

a) In patients with complaints possibly related to previous tick bite(s) and with negative laboratory diagnostic tests for LB and TBE, or previously antibiotic-treated LB, what diagnostic tests are relevant for diagnosing or excluding other TBDs, including tick-borne co-infections?
b) Are there any laboratory tests that can reliably support the diagnosis of persistent LB in spite of recommended standard antibiotic treatment?

## Methods

This systematic review followed the Preferred Reporting Items for Systematic Reviews and Meta-Analyses Protocols (PRISMA-P) guidelines (58). Protocols were developed both for the search and the review process.

### Eligibility Criteria

We divided the work into two parts: (1) laboratory diagnosis of (single) TBDs, and (2) laboratory diagnosis of tick-borne co-infections. In both parts, we performed a systematic literature search and screened through the search results according to predefined selection criteria. In the first scientific literature search, we included references comprising research on adults, young people and children with symptoms of the following infections:

- human granulocytic anaplasmosis *(Anaplasma phagocytophilum*)
- rickettsiosis (*Rickettsia helvetica* or *Rickettsia conorii*)
- neoehrlichiosis (*Neoehrlichia mikurensis*)
- babesiosis (*Babesia* spp.)
- hard tick relapsing fever (*Borrelia miyamotoi*)
- tularemia (*Francisella tularensis*)
- bartonellosis (*Bartonella* spp.)

or with persisting symptoms after antibiotic treatment of LB (“chronic Lyme disease” or “post treatment Lyme disease syndrome”).

All laboratory methods identified in the literature search were considered as relevant, e.g., enzyme-linked immunosorbent assays (ELISA), immunofluorescent assays (IFA), immunoblotting, polymerase chain reaction (PCR), microscopy and culture. The following study designs were included: systematic reviews, cross sectional studies and case control studies. We also included case series and case studies mentioning diagnosis or diagnostic tests in the abstract. The search was limited to the publication years 2007–2017 to focus on more recent methods such as PCR. We excluded studies on tests for the diagnosis of early localized and early/late disseminated LB and TBE. We excluded studies on infections in ticks and domestic or wild animals.

In the second literature search, we included all studies reporting prevalence of or diagnostic methods for identifying co-infections between two or more of the ten infections included in the first search. In addition, we included studies on all stages of LB as well as TBE. This search was also limited to publication years 2007–2017. Studies on patients with other co-infections than TBDs, e.g., HIV, were excluded.

### Information Sources

We searched the following databases: MEDLINE (Ovid), Embase (Ovid), Cochrane Database of Systematic Reviews (Cochrane Library), Database of Abstracts of Reviews of Effects (CRD DARE), Health Technology Assessments Database (CRD HTA), Epistemonikos, ISI Web of Science, Scopus, Prospero, ClinicalTrials.gov, WHO International Clinical Trials Registry Platform (ICTRP). In the first search, all databases mentioned above were searched by Kirkehei in January 2018, and in the second search, Kirkehei searched the following databases in August 2018: MEDLINE (Ovid), Embase (Ovid), Epistemonikos and ISI Web of Science.

### Search Strategy

A research librarian (Kirkehei) performed systematic searches based on the eligibility criteria (Table 1). All searches were described in detail in a separate report from the Norwegian Institute of Public Health (59). Another librarian, the project group at the Norwegian Institute of Public Health and the Nordic group of physicians (hereafter called “the Nordic expert group”) assured the quality of the search strategies.

**Table 1:** Eligibility criteria used for the systematic literature search.

**Part 1–Laboratory diagnosis of tick-borne infections**	
Population*:*	Adults, young people and children with symptoms of the following infections: - human granulocytic anaplasmosis *(Anaplasma phagocytophilum*) - rickettsiosis (*Rickettsia helvetica* or *Rickettsia conorii*) - neoehrlichiosis (Neoehrlichia mikurensis) - babesiois (*Babesia* spp.) - hard tick relapsing fever (*Borrelia miyamotoi*) - tularemia (*Francisella tularensis*) - baronellosis (*Bartonella* spp.) or with persisting symptoms after antibiotic treatment of LB (“chronic Lyme disease” or “post treatment Lyme disease syndrome”)
Diagnostic methods:	All laboratory methods identified in the literature search were relevant, e.g., enzyme-linked immunosorbent assays (ELISA), immunofluorescent assays (IFA), immunoblotting, polymerase chain reaction (PCR), microscopy and culture.
Comparison:	For diagnostic studies: Reference test. All methods were relevant for inclusion.
Outcomes:	Statistical measures of diagnostic performance or test accuracy measures, such as sensitivity/specificity, positive/negative predictive value, likelihood ratios. Studies based on reported clinical outcomes were included.
Study design:	Systematic reviews, cross sectional studies, case control studies. Case series and case studies mentioning diagnosis or diagnostic tests in the abstract were also included.
Exclusion:	Studies on tests for the diagnosis of tick-borne encephalitis (TBE) and early localized and early/late disseminated Lyme borreliosis (LB). Studies on infections in ticks and domestic or wild animals.
**Part 2–Co-infections**	
Inclusion:	All studies reporting prevalence or diagnostic methods for identifying tick-borne co-infections involving microorganisms included in part 1. In addition, studies on all stages of LB and TBE were included.
Exclusion:	Studies on patients with other co-infections than tick-borne diseases, e.g., HIV.

Kirkehei performed the searches in January 2018. The searches consisted of subject headings and free text terms describing the included TBDs and terms typically used when describing diagnostics (for instance diagnostic performance, sensitivity, specificity) or relevant study designs (for instance cross-sectional studies). The first search was limited to studies mentioning “ticks” (and other terms describing tick-bites) in the title or abstract. In a second supplementary search, this limitation was removed. Studies on animals or ticks (without mentioning humans) were also excluded from the search.

### Study Selection

References from the literature search were exported to the online screening tool Covidence (60). Two of the following persons independently screened all references (Kirkehei, Flottorp, Aaberge or Aase), and disagreements were resolved through discussion. The references were screened based on title and abstract, full texts were not read at this stage.

Included references were exported to the reference management system EndNote X9 (Clarivate Analytics, Philadelphia, PA, USA) where one person (Kirkehei) sorted the references into categories by infection type and publication year. The project group at the Norwegian Institute of Public Health checked the final sorting result.

In the first broad search (diagnostic tests), Kirkehei extracted information on diagnostic methods provided only in the abstracts. To ascertain relevance and to assess methodological quality, the Nordic expert group read the studies in full text. At this point, references where only abstract and no full text was available were excluded as well as case reports, case series and papers written in other languages than English. After assessment of the full-text articles, non-systematic reviews and studies of methods not intended for clinical diagnostics in humans were also excluded.

### Data Management

Two reviewers from the Nordic expert group independently extracted data on authors, scientific journal and year of publication, country where the study was conducted, number of participants in study population, type of method that was studied, antigen or target gene used in the studied method, if the index test had been compared with a reference test/standard, diagnostic accuracy (i.e., sensitivity, specificity, negative predictive value, positive predictive value), and study findings. The expert reviewers independently assessed the risk of bias in each individual study. For the assessment of diagnostic studies, the QUADAS (61) checklist was used, whereas the AMSTAR (62) checklist was applied for systematic reviews. Each study obtained an over-all classification of high, medium, or low risk of bias. Disagreements between the reviewers were discussed and resolved through consensus or, if needed, by an extra expert reviewer. In some cases, a risk classification of low/medium or medium/high were considered appropriate. In case a reviewer had co-authored an article, the review task was given to another independent reviewer.

### Summarizing Results

A descriptive analysis stratified by each TBD was used to summarize studies included in this systematic review. Themes for analysis included types of diagnostic methods, test performance, applicability, relevance and usefulness in clinical practice.

## Results

### Study Selection

**Figure 1:**
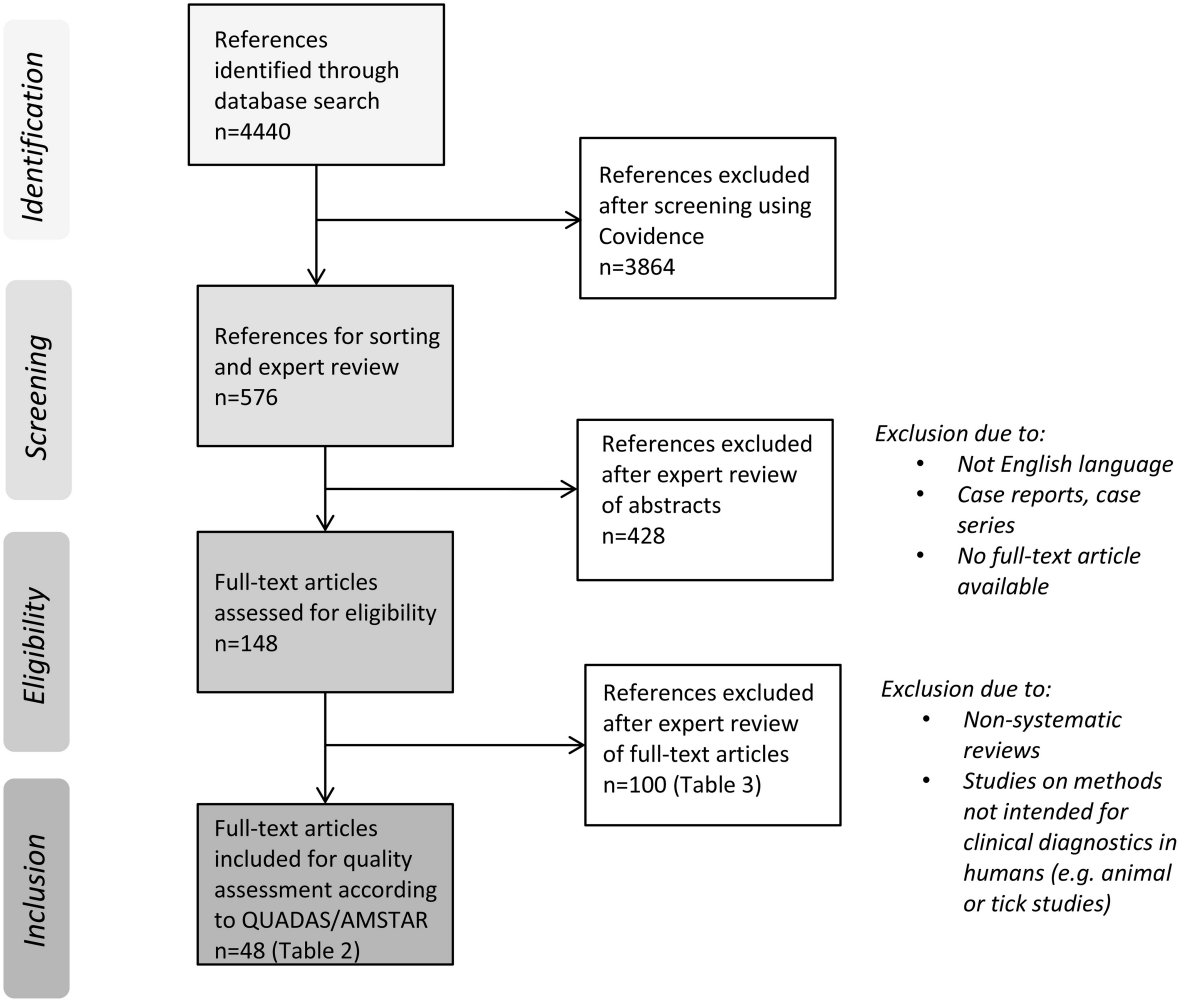
Flow diagram of literature search and study selection process.

The study selection process and reasons for exclusion are shown in Figure 1. The search retrieved 4, 440 unique references. A total of 3, 864 references were excluded through an initial screening of the titles and/or the abstracts by two independent persons using Covidence as described above. We included 576 references and sorted them according to the type of TBD. One hundred forty-eight full-text articles were assessed for eligibility by the expert reviewers; 48 were included for quality assessment according to the QUADAS or AMSTAR checklists (Table 2). References that were excluded at this point are listed in Table 3. The results of the in-depth expert review (QUADAS/AMSTAR) are summarized below and in Table 2.

**Table 2:** General information on the 48 publications that were included for quality assessment according to the QUADAS (diagnostic studies) or AMSTAR (systematic reviews) checklists.

**Human granulocytic anaplasmosis (** ***Anaplasma phagocytophilum*** **)**							
**Author, publication year, journal**	**Country, No of study participants**	**Type of diagnostic method**	**Type of antigen, target gene, etc**	**Reference test/ reference standard**	**Diagnostic accuracy**	**Risk of bias**	**Comments/conclusions**
Pan et al., 2011, J Clin Microbiol	China, 42	Loop-mediated isothermal amplification (LAMP)	msp2	Either 4-fold increase of antibody titer or positive nested PCR targeting 16SrRNA gene or positive real-time PCR targeting msp2	Sensitivity 62%, specificity 100%	Medium	Selected patient group, all were either seropositive or PCR-positive in another laboratory before inclusion
Schotthoefer et al., 2013, J Clin Microbiol	USA, 361	Real-time PCR	groEL	Blood smear	Sensitivity 100%, specificity not evaluated	Low	Clinically relevant study population, medical charts reviewed and clinical assessment before index test. Due to lack of proper reference test sensitivity could only be compared to blood smear in early acute phase of disease. Serology could not be evaluated due to lack of paired serum samples. Test results were presented without performance evaluation. Conclusion that PCR is better than blood smear in acute phase, serology better than PCR in late phase (> 4 days).
Sanchez et al., 2016, JAMA	USA, 361 articles reviewed in depth					Low (no statistical methods used)	Systematic review. Short paragraph on laboratory diagnostics of A. phagocytophilum stating that microscopy on blood smear or buffy coat, PCR of blood and/or serologic testing may be used, evidence grading I-B for all three methods (American Evidence-Based Scoring System) This review also included laboratory diagnosis of babesiosis.
**Rickettsiosis (** ***Rickettsia helvetica, Rickettsia conorii*** **)**							
Boretti et al., 2009, Appl Environment Microbiol	Germany/Switzer-land, 884 dogs, 58 foxes, 214 humans, 2073 ticks	Real-time PCR	23S rRNA *(Rickettsia helvetica-*specific*)*	*glta* PCR (Stenos, gltA gene)	Sensitivity 75% positive in dilution 1-10 copies/mL, Specificity 100%	Low/ medium	Stenos PCR was used to confirm the presence of Rickettsiae. The human samples were anonymous – spectrum bias?
Mouffok et al., 2011, Emerg Inf Dis	Algeria, 39 patients, 41 swab samples	qPCR + *Rickettsia conorii*-specific qPCR	RC0338 gene + acetyl-transferase gene in *Rickettsia conorii*-specific qPCR	qPCR coding β-actin (Raoult 2011)	Sensitivity 63.4%, Specificity 100%	Low	The clinical picture was judged as rather typical. Difficult to determine quality and bias.
Renvoise et al., 2012, FEMS Immunol Med Microbiol	France, 465 patients, 643 samples	qPCR	Probes for SFG, TG *Rickettsia* and *Rickettsia* spp. Hypothetical protein (RC0338 gene)	Conventional PCR and sequencing, WB	Methodological sensitivity: 1 bacterium, specificity 100%	Medium/ high	Short communication, scarce details.
Kowalczewska, 2012, FEMS Immunol Med Microbiol	France, 48 patients (10 *Rickettsia typhiii*, 28 *Rickettsia conorii*, 10 blood donors)	Serology (ELISA)	60 kD, Sca1, Ad2, omp1, pepA, RP631, spo01, 3-methylubi-quinone-9, 3-methyltransfer-ase, UDP-, Signal protein, FOF1, VapC1, VapB1, PLD, Sca13, Sca10, Dihydrofolate reductase, Hypothetical protein, RickA, Tu,	IFA + real-time PCR	Sensitivity 0-70%, Specificity 90-100%	Medium	Short communication. Few patients in every group.
Do et al., 2009, Microbiol Immunol	Korea, 136 sera	In-house ELISA	Recombinant OmpA and OmpB antigen from *Rickettsia conorii*	Commercially available ELISA kit with whole OmpA and OmpB antigens from *Rickettsia conorii*	Recombinant OmpA: sensitivity 90%, specificity 100%. Recombinant OmpB: sensitivity 90-95%, specificity 95-100%	Low	The data suggest that the recombinant antigens have high specificity for *Rickettsia conorii*.
Kantsö et al., 2009, J Microbiol Methods	Denmark, 111 Weil Felix (WF)-sera + 106 blood donor sera =total 217	Two IFA methods (A and B)	Whole-cell bacteria IFA A - *Rickettsa rickettsii, Rickettsia typhii* IFA B -*Rickettsia typhii, Rickettsia rickettsii, Rickettsia conorii, Rickettsia helvetica*	WF	WF vs IFA A: sensitivity 74%, specificity 79%. WF vs IFA B: sensitivity 60%, specificity 73%. IFA A vs IFA B: WF titer >200, 100% concordance. IFA A vs IFA B: WF titer 25-50, 38% and 56% concordance, respectively. IFA A vs IFA B: WF titer <25, 5% and 68% positives, respectively.	Low	
Khrouf et al., 2015, Ticks Tickborne Dis	Tunisia, 101 patients, 121 samples	Reverse line blot (RLB)	23S-5S rRNA gene	qPCR	Sensitivity 46.4%, specificity 86.1% (kappa value 0.33)	Low/ medium	
Znazen et al., 2015, PLoS ONE	Tunisia, 180 patients (180 sera, 174 blood samples, 77 biopsies)	qPCR 1 (all *Rickettsiae*) and 2 (spotted fever group)	Several sequences including 16sRNA gene	MIF	Serology positive in 82/183 (45%). qPCR positive in 46/182 (56%). qPCR diagnostic sensitivity (5%)-47.7%-54.5%%, specificity 100%. Methodological sensitivity = 2 copies/reaction for all PCRs (Rsp, Rtt, RCO338, Rp278)	Low	Differences in diagnostic sensitivity depending on test material. However the patients were judged having a rickettsial infection based on serology, but we do not know if there were rickettsial bacteria in the samples. Positive serology was used for defining diagnosis. Improved sensitivity with qPCR in skin biopsies vs whole blood samples and in initially seronegative patients. Some of the patients had taken antibiotics before analysis.
Bizzini et al., 2015, Microbes and Infection	France, 213 sera (63 Q-fever; 20 spotted fever; 6 murine typhus; 124 controls)	Epifluor-escence immunoassay (InoDiag)	*Rickettsia typhii, Rickettsia conorii, Rickettsia felis, Coxiella burnettii*	MIF 1 (*Coxiella burnettii* phase 1 och 2-antigens) and MIF 2 (*Rickettsia conorii, Rickettsia typhi, Rickettsia africae* antigens)	Sensitivity Q fever: acute Q fever): 20-30% (IgG),75- 83% (IgM), chronic Q-fever: 100%, past Q fever: 48-63% Sensitivity Spotted MSF/murine typhus - 91-100% Specificity Q fever: 82-100% Specificity Spotted MSF/murine typhus: 79-98%	Low	Few cases per diagnosis.
**Neoehrlichiosis (** ***Neoehrlichia mikurensis*** **)**							
Qarsten et al., 2017, Ticks Tick Borne Dis	Norway, 70 patients with symptoms after tick bite	Commercial multiplex PCR and singleplex real-time PCR	Real-time PCR: groEL Multiplex PCR: not specified in article, only: ”Specific probes directed against…*Ehrlichia* (*Ca*. N. mikurensis, *E. chaffeensis* and *E. ewingii*)”	None	Commercial multiplex PCR: 4/69 (6%) positives, real-time PCR 7/70 (10%) positives	Low	The commercial multiplex PCR bacteria flow chip system failed to identify half of the infected patients detected by corresponding real-time PCR protocols. The recovery of *Ca*. N. mikurensis DNA was higher in the pellet/plasma fraction of blood than from whole blood.
**Babesiosis (** ***Babesia*** **spp)**							
Duh et al., 2007, Parasitology	Slovenia, 7 ; Austria, 2	IFA	*Babesia microti* + *Babesia divergens*	Blood smear microscopy + PCR	Not applicable	High	Only 10 serum samples, patient samples (n=9, “history of tick bite”) and one sample from Fullerlabs. No negative controls. Unclear which analysis were performed on which samples. There were too few patients to calculate diagnostic accuracy.
Ohmori et al., 2011, Parasitology Int	Japan, 8	PCR	4 genotype-specific (Kobe, Otsu, Nagano, US-type)	Blood smear microscopy and/or IFA	Not applicable	High	One patient, one asymptomatic positive blood donor and 7 negative controls. Not described how the patient or the positive blood donor were confirmed positive. There were too few patients to calculate diagnostic accuracy.
Priest et al., 2012, Clinical & Vaccine Immunology	USA, 236 + Haiti, 30	Multiplex IgG assay	BMN1-9/BmSA1-antigen	Blood smear microscopy + IFA	Sensitivity 97.4%, specificity 97.6%	Medium/high	Patient samples from CDC investigated for malaria and babesiosis and a negative control group. Unclear if the negative control group were investigated by blood smear.
Teal et al., 2012, J Clin Microbiol	USA, 40 (+671)	Real-time PCR	*Babesia microti* 18S rRNA	Blood smear microscopy + conventional PCR	Sensitivity 5-10 parasites/μl, specificity 100%	Medium	Patients analysed for parasite infections. Real-time PCR compared to microscopy and conventional PCR with the aim of replacing conventional PCR with real-time PCR. Real-time PCR more sensitive than Giemsa stain.
Rollend et al., 2013, Vector Borne & Zoonotic Dis	USA, 19	PCR	*Babesia microti* 18S rRNA (BabMq18)	Blood smear microscopy	Sensitivity 100%, specificity 100%	Medium/high	14 patients with babesiosis and 5 healthy controls. The method only detects *B. microti*. Unclear if all samples were analyzed with blood smears.
Levin et al., 2014, Transfusion	USA, 74 (+ 1003 +15 000 blood donors)	EIA	BMN1	IFA + PCR + blood smear microscopy	Sensitivity 88%, specificity 99.5%	Medium	Evaluated with regards to patient samples, not blood donors. Unclear if all three methods were performed on all samples.
Wang et al., 2015, Diagnostic Microbiol Infect Dis	USA, 36	PCR	*Babesia microti* 18S rRNA	Blood smear microscopy and serology	Not applicable	High	It is not clear from the article which analyses were made on each sample.
Racsa et al., 2015, J Clin Microbiol	Texas USA, 281 (6 Babesia spp, 275 Plasmodium spp)	CellaVision (digital hematology analyzer)	Microscopy	Blood smear conventional microscopy	Sensitivity 100%, specificity 100%	High	Only 6 samples positive for *Babesia* spp. were included, the rest were malaria samples.
Wang et al., 2015, Ticks Tick-borne Dis	USA, 152	PCR	*Babesia microti* 18S rDNA	Blood smear microscopy	Sensitivity 100%, specificity 97.7%	Low	Patient samples sent for parasite analysis. PCR and blood smear performed on all samples.
Chen, 2016, PLos Neglected Tropical Dis	China, 100 healthy controls but number of patients not clearly stated	PCR	*Babesia microti, Babesia divergens, Babesia duncani, Babesia venatorum* 18S rDNA	Blood smear microscopy	Sensitivity 100%, specificity 97.0% for *Babesia microti*, specificity 97.9% for *Babesia venatorum*	High	Patient group not clearly defined in the method section. In the article the authors state that they included patients with fever but not how many, only the total number of samples which includes animal and vector samples.
Aase et al., 2016, Infectious Diseases	Norway, 62 (21 patients + 41 controls)	Modified microscopy protocol (”LM method”)	Direct microscopy	Conventional microscopy, PCR and serology	Not applicable	Low	The structures interpreted as Borrelia and Babesia by the LM-method could not be verified by PCR. Because of this, diagnostic accuracy could not be calculated.
Levin et al., 2016, Transfusion	USA, 129 (+ 26 703 blood donors)	EIA	BMN1-9/BmSA1-antigen + BMN1-17	IFA + PCR + blood smear microscopy	Sensitivity 84.5%	Medium	Unclear how many of the 129 patients were diagnosed with blood smear microscopy or PCR.
Hanron et al., 2017, Diagnostic Microbiol Infect Dis	USA, 18	PCR	*Babesia microti* 18S rRNA	18S rDNA	Not applicable	High	Reverse transcription PCR much more sensitive than PCR. It is unclear from the article which was the reference test, diagnostic accuracy could not be calculated. Few number of positive samples.
Souza et al., 2016, American Journal Tropical Medicine Hygiene	USA, 78	4 different real-time PCR methods and nested PCR	*Babesia microti* 18S rRNA	Blood smear	Sensitivity 100%, specificity 100%	Low	Sensitivity and specificity varied between the different real-time PCR methods from 71% to 100% (CI 95%)
Sanchez et al., 2016, JAMA	USA, 361 articles reviewed in depth					Low (no statistical methods used)	Systematic review. Microscopy on thin blood smear, evidence grading I-B (American Evidence-Based Scoring System). PCR should be considered early in the infection when parasites are few, but should be used with caution when monitoring response to therapy since DNA can be detected for a long time after parasites are no longer visualized in blood smears (IIb-B). Serology can confirm the diagnosis (I-B), but cannot replace microscopy and PCR. This review also included laboratory diagnosis of anaplasmosis.
**Hard tick relapsing fever (** ***Borrelia miyamotoi*** **)**							
Lee et al., 2014, Int J Mol Sci	USA, 14	Nested PCR and direct Sanger DNA sequencing	16SrRNA	Method tested in a group of patients with clinically suspected LB, no *Borrelia miyamotoi* reference test/standard	Not applicable	High	PCR method developed and extraction method optimized using cultured *Borrelia burgdorferi* sensu stricto strain B31 and *Borrelia myiamotoi* DNA extracted from ticks. The method was then used to test EDTA plasma from 14 patients with clinically suspected LB without specification of diagnostic criteria. All patient samples were positive for *Borrelia burgdorferi* or/and *Borrelia miyamotoi*. No reference standard used and diagnostic accuracy cannot be assessed.
Molloy et al., 2017, Clin Infect Dis	USA, 30 (24 were evaluable)	ELISA	C6	PCR	Overall sensitivity 91.7%. Acute phase sensitivity (<6 days) 16.7%. Convalescent phase (> 6 days) 86.7% Specificity not evaluated (C6 ELISA originally designed to diagnose LB)	Medium	The patients tested were pre-selected and all of them were PCR-positive for *Borrelia miyamotoi*. Sensitivity may therefore be overestimated.
Koetsveld et al., 2017, CMI	Russia, 9	Culture	Modified Kelly-Pettenkorfer medium with 10% fetal calf serum	PCR	Not applicable	High	The aim of the study was to optimize culture procedures in order to retrieve clinical isolates for future research, not for clinical diagnostic use (too slow compared to PCR, less sensitive). All included patient samples were PCR positive, and few samples were available. Sensitivity/specificity cannot be evaluated.
Jahfari et al., 2017, J Microbiol Methods	Russia, 84	Luminex	recombinant GlpQ	PCR	Sensitivity IgM 54%, IgG 38%, IgM+IgG 69%, Specificity IgM 98%, IgG 92%	Medium-High	The aim was to validate a recombinant GlpQ assay for clinical laboratory diagnostic use. A case-control design was used which may have over-estimated the diagnostic accuracy.
**Tularemia (** ***Francisella tularensis*** **)**							
Gouriet et al., 2008, Clin Microbiol Inf	France, 248	Serologic multiplex array	Whole cell	IFA	IgG 100/95 sens/spec IgM 100/100 sens/spec in 16 patients	High	Selected material, patients with pneumonia
Splettstoesser et al., 2010 J Clin Microbiol	Germany, 58 healthy + 58 tularemia patients	Serology (ICT)	LPS and whole cells	MAT	ICT sensitivity 98.3%, specificity 96.5%	Medium	Highly selected material for comparison of 2 antibody assays.
Kilic et al., 2012, Dg Microbiol Inf Dis	Turkey, 345 109 tularemia cases, 236 healthy or other infections	Serology (ICT)	LPS and whole cells	MAT	ICT sensitivity 99.3%, specificity 94.6%	Medium	Antibody assay comparison in historical material
Sharma et al., 2013, Clin Vaccin Immunol	Japan, 69	Serology (competitive ELISA)	LPS and whole cells	MAT and indirect ELISA	Competitive ELISA sensitivity 91.1%, specificity 97%. Indirect ELISA sensitivity 94.1%, specificity 98%. MAT sensitivity 81.8%, specificity 98%.	Medium	Antibody assay comparison in serum samples from 19 tularemia patients and 50 healthy individuals.
Chaignat et al., 2014, BMC Infect Dis	Serbia, 204	Serology (2 commercial ELISAs, 1 in-house ELISA, 1 ICT, 1 in-house antigen microarray, 1 WB	LPS and whole cells	MAT	Sensitivity/specificity for Serion ELISA IgG 96.3%/96.8% Serion ELISA IgM 94.9%/96.8% Serazym ELISA 97%/91.5% In-house ELISA 95.6%/76.6% VIRapid ICT 97%/84% In-house microarray 91.1%/97.9%	Medium	Case-control
Cubero et al., 2018, EurJ Clin Microbiol Inf Dis	Spain, 773 (364 diagnosed with tularemia)	Serology (commercial chemi-luminescence test)	Virclia CHT IgM/G	MAT, ICT, in-house ELISA IgG, and IgM.	Clinical diagnostic sensitivity 91.8%, specificity 96.7%.	Medium	Case-control. Performance similar to reference tests.
Yanes et al., 2018, J Clin Microbiol	France, 208	Serology (1 commercial ELISA, 1 commercial ICT)	ELISA IgM and IgG: LPS ICT: n.a.	In-house MAT and IFA	ELISA: IgM Sensitivity 88.2%, specificity 94.8%; IgG Sensitivity 86.3%, specificity 95.5%. ICT: IgM/IgG Sensitivity 90%, specificity 83.6%	Medium	Cross sectional and case control study design combined.
**Bartonellosis (** ***Bartonella*** **spp)**							
Maggi et al., 2011, Diagn Micriol Inf Dis	USA, 192	PCR 'bacteremia'	Culture enrichment	Non enrichment	Enrichment > non-enrichment Serology positive in 49.5 % PCR positive in 23.9 %	High	Laboratory cross sectional study of PCR detection of Bartonella spp compared to observed seropositivity
Tsuruoka et al., 2012, Diagn Microbiol and Inf Dis	Japan, 206	Serology (ELISA)	N-lauroyl-sarcosine soluble protein	IFA	ELISA sensitivity 95.7%, specificity 97.7%	Medium	Laboratory case-control assay comparison.
Smit et al., 2013, Am J trop med and hyg	Peru, 65	qPCR experimental	Dried blood spots Bartonella bacilliformis	Blood smear microscopy	PCR > smear	High	Low number of detected infections, 3% blood smear, 24.6% PCR
Pultorak et al., 2013, J Clin Microbiol	USA, 91	PCR, culture enrichment	Sequential testing for 1 week	n.a.	2-3 PCR > 1PCR	High	Retrospective
Vermeulen et al., 2007 Clin Microbiol Infect	The Netherlands, 107	In-house serology (IFA) IgM and IgG	Whole cells (*B. henselae* ATCC 49882 = *B. henselae* type Houston-1)	PCR targeting the 16S rRNA gene	IFA IgM/IgG sensitivity 53%/67%, specificity 93%/82%. ELISA IgM/IgG sensitivity 65%/ 28%, specificity 91%/91%	Medium	The serological assays evaluated indicated low sensitivity, thus inappropriate as rule out tests for cat scratch disease.
Caponetti et al., 2009, Am J Clin Path	USA, 38	IHC	*B. henselae* monoclonal antibody; clone H2A10	None	n.a.	High	Diagnostic sensitivity in evaluated tests including IHC is low for cat scratch disease. PCR and Steiner Silver stain were also performed and authors conclude that diagnostic sensitivity is low for all three tests (25-46% positives among cases with histologically or clinically suspected CSD).
Vermeulen et al., 2010, J Med Microbiol	The Netherlands, 105	Serology (5 IFA, 1 ELISA)	Houston or Marseille strains	Lymphadeno-pathy + positive PCR targeting the 16S rRNA gene, and exclusion of other causes of lymphadeno-pathy	Sensitivity IgM 50-62%, specificity IgM 87-96%. Sensitivity IgG 88-98%, specificity IgG 69-89%.	High	The study confirms difficulties with the serodiagnosis of cat scratch disease using in-house and commercial tests.
Kawasato et al., 2013, Rev Inst Mad Trop Sao Paulo	Brazil, 18	Three PCR assays	60 kD heat schock protein (HSP), FtsZ, 16S-23S intergenic spacer	None	The nested-FtsZ was more sensitive than nested-HSP and nested-ITS (p <0.0001), enabling the detection of Bartonella henselae DNA in 15 of 18 patients (83.3%).	High	Small methodological study.
Otsuyma et al., 2016, J Clin Microbiol	Japan, 132 (24 definite and 23 suspected bartonellosis cases)	Serology (IgM ELISA vs IgM IFA)	N-lauroyl-sarcosine-insoluble proteins	Whole cell IgM IFA	Sensitivity ELISA 49-64%, IFA 28%	Medium	Laboratory method development.
Tsuneoka et al., 2017, Diagn Microbiol and Inf Dis	Japan, 100 clinically suspected CSD cases and 90 healthy controls	Serology (conventional IFA vs strain-specific IgM IFA)	Strain-specific antigen	Whole cell IFA	15 of suspected cases were positive with conventional IFA, 21 were positive with strain-specific IgM IFA	High	The strain-specific IFA greatly improved the accuracy of diagnosis, thus better diagnostic accuracy is achieved if antigens from country-specific strains are used.
**Tick-borne co-infections (multiple tick-borne microorganisms)**							
No studies fulfilled the inclusion criteria.							
**Persistent post-treatment Lyme borreliosis (** ***Borrelia burgdorferi*** **sensu lato)**							
No studies fulfilled the inclusion criteria.							

*PTLDS, post-treatment Lyme disease syndrome; MRI, magnetic resonance imaging; CSF, cerebrospinal fluid; rCBF, regional cerebral blood flow; PET, positron emission tomography; LB, Lyme borreliosis; WB, western blot; ELISA, enzyme-linked immunosorbent assay; WCS, whole cell sonicate; GlpQ, glycerophosphodiester-phosphodiesterase; IFA, immunofluorescence assay; WF, Weil-Felix; RLB, reverse line blot; MIF, micro-immunofluorescence; EIA, enzyme immune assay; LPS, lipopolysaccharide; ICT, immunochromatographic test; n.a., not applicable; IHC, immunohistochemistry; CDC, Centers for Disease Control and Prevention; CSD, cat scratch disease; MAT, micro-agglutination test. One publication, Sanchez et al., 2016, JAMA, covered both anaplasmosis and babesiosis and is therefore presented twice in the table*.

**Table 3:** Full-text publications reviewed but excluded from further quality assessment by QUADAS/AMSTAR.

**Author, publication year, journal**	**Reason for exclusion**
**Human granulocytic anaplasmosis (** ***Anaplasma phagocytophilum*** **)**	
Al-Khedery et al., 2014, Pathogens	Do not describe a method for clinical diagnostics in humans, but rather a method for epidemiological surveillance of *A. phagocytophilum* in ticks.
Silaghi et al., 2017, Vector Borne Zoonotic Dis	Not systematic review.
Cooper et al., 2015, Clinical Microbiology Newsletter	Not systematic review.
Bakken et al., 2015, Infect Dis Clin North Am	Not systematic review.
Atif et al., 2015, Parasit Res	Not relevant, review of ecology and epidemiology.
Schotthoefer et al., 2014, Wmj	Not systematic review.
Jin et al., 2012, Vector Borne Zoonotic Dis	Not systematic review (despite the title the method is not described and cannot be assessed).
Rymaszewska et al., 2011, Veterinarni Medicina	Not relevant, epidemiologic study on dogs.
Bakken and Dumler, 2008, Infect Dis Clin North Am	Not systematic review.
Dhand et al., 2007, Clin Inf Dis	Not systematic review.
Eshoo et al., 2010, J Clin Microbiol	Not relevant, diagnostic performance evaluated only for Ehrlichia spp. and *Ehrlichia chaffeensis*
Ismail et al., 2010, Clin Lab Med	Not systematic review.
Bitam and Raoult, 2009, Curr Probl Dermatol	Not systematic review.
Dana et al., 2009, Dermatologic Therapy	Not systematic review.
**Rickettsiosis (** ***Rickettsia helvetica, Rickettsia conorii*** **)**	
Biggs HM et al., 2016, CDC report	Not systematic review.
Rahdi M et al., 2015, Indian J of Medical Research	Not systematic review.
Paris DH et al., 2016, Curr Opin Infect Dis	Not systematic review.
Chanana L et al., 2016, J Glob Infect Dis	Not systematic review.
**Neoehrlichiosis (** ***Neoehrlichia mikurensis*** **)**	
Wenneras C et al., 2017, Inf Dis	Study of risk factors for neoehrlichiosis. Diagnostic performance was not assessed.
Silaghi C et al., 2016, Exp Appl Acarol	Not systematic review.
**Babesiosis (** ***Babesia*** **spp.)**	
Simonetti et al., 2016, Transfusion	Not relevant. Blood donors, not patient samples. Model for risk assessment, not patients.
Bish et al., 2015, Transfusion	Not relevant, model for calculating cost effectiveness for screening program for blood donors.
Gabrielli et al., 2012, Vector Borne Zoonotic Dis	Not relevant, no patients with symptoms.
Rozej-Bielicka et al., 2017, Parasitology Res	Not relevant, not patients with symptoms (only asymptomatic individuals).
Wilson et al., 2015, Exp Parasitol	Not relevant, not humans (hamsters).
Verma et al., 2015, Am J Trop Med Hyg	Not relevant, no patient samples. Only mouse models/molecular biology not related to humans.
Leiby et al., 2014, Transfusion	Not relevant, asymptomatic individuals with previous positive serology.
Imugen, 2011 Clinical Trial	Not relevant, no patient samples, only blood donors.
Edappallath et al., 2017, Transfusion	Not systematic review.
Saleh et al., 2015, J Egypt Soc Parasitol	Not systematic review.
Parija et al., 2015, Trop Parasitol	Not systematic review.
Ord and Lobo, 2015, Curr Clin Mibrobiol Rep	Not systematic review.
Hildebrandt et al., 2013, Infection	Not systematic review.
Vannier and Krause, 2012, N Engl J Med	Not systematic review.
Shah et al., 2012, Europ Infect Dis	Not systematic review.
Vannier and Krause, 2009, Interdiscip Perspect Infect Dis	Not systematic review.
Vannier et al., 2008, Infect Dis Clin North Am	Not systematic review.
Blevins et al., 2008, Cleve Clin J Med	Not systematic review.
**Hard tick relapsing fever (** ***Borrelia miyamotoi*** **)**	
Sinski et al., 2016, Adv Med Sci	Not systematic review.
Telford et al., 2015, Clin Lab Med	Not systematic review.
Krause et al., 2015, Clin Microbiol Infect	Not systematic review.
**Tularemia (** ***Francisella tularensis*** **)**	
Banada et al., 2017, J Clin Microbiol	Not systematic review
Seo et al., 2015, Biosens Bioelectron	Not systematic review
Seiner, 2013, J Appl Microbiol	Not systematic review
Matero, 2011, Clin Microbiol Infect	Not systematic review
Janse, 2010, BMC Microbiol	Not systematic review
Jiang, 2007, Anal Chim Acta	Not systematic review
Rastawicki, 2015, J Microbiol Methods	Not systematic review
Zasada, 2015, Lett Appl Microbiol	Not clinical
Janse, 2012, Plosone	Not clinical
Buzard, 2012, Forensic Sci Int	Not clinical
Dauphin, 2011, Diagn Microbiol Infect Dis	Not clinical
Mitchell, 2010, Mol Cell Probes	Not clinical
Molins, 2009, Diagn Microbiol Infect Dis	Not clinical
**Bartonellosis (** ***Bartonella*** **spp.)**	
Liu et al., 2017, J Microbiol Meth	Methods not evaluated on clinical samples.
Ferrara et al., 2014, Lett Appl Microbiol	Experimental serology, not clinical.
Smit et al., 2013, Am J Trop Med	Experimental PCR, not clinical.
Pultorak et al., 2013, J Clin Microbiol	Experimental PCR enrichment preculture, not clinical.
Bergmans et al., 2013, Meth in Mol Biol	Experimental PCR, not clinical.
Abarc et al., 2013, Rev Chilena Meth	Experimental serology, not clinical.
Saisonkorh et al., 2012, FEMS Microbiol Lett	Experimental proteomics, not clinical.
Tang et al., 2009, J Clin Microbiol	Experimental PCR, not clinical.
Hoey et al., 2009, CVI	Laboratory comparison of serologic methods, not clinical.
Fournier et al., 2009, J Med Microbiol	Experimental MALDI-TOF, not clinical.
Wagner et al., 2008, Int J Med Microbiol	Laboratory comparison of serologic methods, not clinical.
Sanchez Clemente et al., 2012, PLoS Negl Trop Dis	*Bartonella bacilliformis*, not present in Europe.
Angkasekwinai et al., 2014, Am J Trop Med	Not clinical.
Gutierrez et al., 2017, Vector Borne & Zoonotic Dis	Not systematic review.
Breitchwerdt et al., 2017, Vet Dermatol	Not systematic review.
Amer et al., 2017, Curr Opin Ophtalmol	Not systematic review.
Bonhomme et al., 2008, Curr Immunol Rev	Not systematic review.
Bloch et al., 2007, Curr Infect Dis Rep	Not systematic review.
**Tick-borne co-infections (multiple tick-borne microorganisms)**	
Angelakis, 2009, European Journal of Clinical Microbiology and Infectious Diseases.	No tick association.
Schlachter, 2017, Methods in Molecular Biology	Method development. No evaluation on clinical samples.
Chan, 2013, BMC Microbiology	Method development. No evaluation on clinical samples.
Source, 300 Antibody Diagnostic Test Kit. Ongoing clinical trial	No tick association.
Jensen, 2017, Ugeskrift for Laeger	Not systematic review.
Eickhodd, 2017, Cleveland Clinic Journal of Medicine	Not systematic review.
Sanchez, 2016, Journal of the American Medical Association	No evaluation on clinical samples.
Choi, 2016, Current Sports Medicine Reports	Not systematic review.
Nathavitharana, 2015, Clinical Medicine	Not systematic review.
Schmitt, 2012, Infectious Disease Clinics of North America	No tick association.
Dana, 2009, Dermatology Therapy	Not systematic review.
Bitam & Raoult, 2009, Current Problems in Dermatology	Not systematic review.
**Persistent post-treatment Lyme borreliosis (** ***Borrelia burgdorferi*** **sensu lato)**	
Aalto A et al., 2007. Acta Radiol	No laboratory method evaluated.
Fallon BA et al., 2014, Clin Infect Dis.	Degree of inter-laboratory variability was assessed.
Lantos PM et al., 2014, Clin Infect Dis.	Systematic review, but no diagnostic test was evaluated.
D 'Alessandro M et al., 2017. Curr Infect Dis Reports.	Not systematic review.
Nemeth J et al., 2016. Swiss Medical Weekly	Not systematic review.
Halperin JJ, 2016. Acta Neurol Belgica.	Not systematic review.
Halperin JJ, 2015. Inf & Drug Res.	Not systematic review.
Cieszka J et al., 2015. Reumatologia	Not systematic review.
Aucott JN, 2015. Infect Dis Clin North Am.	Not systematic review.
Borgermans L et al., 2014. Int J Family Med.	Not systematic review.
Nichols C, Windermuth B. J for Nurse Practitioners.	Not systematic review.
Ljöstad U et al., 2013. Acta Neurol Scand.	Not systematic review.
Rupprecht TA et al., 2011. Future Neurol.	Not systematic review.
Stricker RB et al., 2008. Future Microbiol.	Not systematic review.
Hoppa E et al., 2007. Curr Opinion in Pediatrics.	Not systematic review.
Feder HM et al. 2007. N Engl J Med.	Not systematic review.

### Human Granulocytic Anaplasmosis (*Anaplasma phagocytophilum*)

Regarding laboratory methods evaluated for diagnosis of human granulocytic anaplasmosis (HGA), two studies on molecular detection (real-time PCR and loop-mediated isothermal amplification) vs. serology or blood smear microscopy and one systematic review were assessed according to the checklists.

### Rickettsiosis (*Rickettsia helvetica, Rickettsia conorii*)

Nine studies were reviewed, five regarding molecular detection and quantification (PCR, qPCR), of which one compared reverse line blot hybridization vs. qPCR. Four were serological studies [IFA, Western blot (WB), ELISA], one of which compared an epifluorescence immunoassay vs. conventional IFA and another compared ELISA vs. IFA.

### Neoehrlichiosis (*Neoehrlichia mikurensis*)

One study using PCR for laboratory diagnosis of neoehrlichiosis in humans fulfilled the inclusion criteria for publications evaluating diagnostic tests and was reviewed according to the QUADAS checklist. Another publication did not contain information about diagnostic performance and one review was not systematic, and thus, these publications were excluded (Table 3).

### Babesiosis (*Babesia* spp.)

For *Babesia* spp., 14 studies fulfilled the criteria for review according to the QUADAS checklist, eight studies on PCR, four on serology (IFA, multiplex IgG and EIA), one on CellaVision and one on modified microscopy. One systematic review was also included.

### Hard Tick Relapsing Fever (*Borrelia miyamotoi*)

For *B. miyamotoi*, four studies fulfilled the criteria for in-depth review; two studies on serological methods (ELISA and Luminex), one on nested PCR and one aiming primarily at optimizing culture procedures from clinical samples.

### Tularemia (*Francisella tularensis*)

Seven diagnostic studies regarding *F. tularensis* qualified for review according to the QUADAS protocol; all of them on serological methods (ELISA, immunochromatography and Western blot).

### Bartonellosis (*Bartonella* spp.)

Out of 33 abstracts, ten diagnostic studies were included for further review. Five studies presented evaluations of serologic assays (ELISA, IFA), one of immunohistochemistry, and four studies of PCR methods.

### Tick-Borne Co-infections (Multiple Tick-Borne Microorganisms)

Two publications (Schlachter, Chan, Table 3) from the same group of researchers described the same multiplex PCR assay targeting *Borrelia* spp. (recA gene), *A. phagocytophilum* (APH1387 gene) and *Bab. microti* (BmTPK gene). Human blood spiked with cultured *B. burgdorferi* and plasmids containing the target genes from *A. phagocytophilum* and *Bab. microti* added to the extracted DNA were used for developing the method but it was not evaluated on clinical patient samples, and the studies were therefore not included in the review. No other studies fulfilled the inclusion criteria.

### Persisting Post-treatment Lyme Borreliosis; “Chronic Lyme Borreliosis” (*Borrelia burgdorferi* Sensu Lato)

None of the published articles assessed for eligibility (*n* = 16) met the inclusion criteria and all were consequently excluded from the review. No laboratory method useful for clinical diagnostic support of persisting post-treatment LB symptoms was found in this present systematic review. Five publications were primarily included, but later excluded (Table 3). Two out of five publications did not study any laboratory method and one did not focus on persisting LB after antibiotic treatment. In the last two studies, one focusing on serologic response and the other being a review on culture, the authors found the methods not useful for supporting persistent *B. burgdorferi* s.l. infection.

## Discussion

In this systematic review we performed a broad, thorough, and systematic literature search in an attempt to identify all studies mentioning diagnostic methods of TBD, regardless of study design. Nonetheless, we may still have lost some relevant studies. We limited the search to studies mentioning tick or tick bite in the title or abstract. However, not all studies on TBD explicitly mention “ticks,” and therefore we performed a supplementary search without this limitation. Instead, we limited the search to those described as cross-sectional studies or diagnostic accuracy studies. This supplementary search gave some additional references, mainly about diagnostics of tularemia and babesiosis. Due to the study design criteria applied in the supplementary search, we may have missed some relevant publications, i.e., case reports and case series. On the other hand, a major aim of this review was to investigate to what extent the different diagnostic methods described or mentioned in the scientific literature have been evaluated in comparative studies using authentic human clinical samples. The search for Lyme disease (borreliosis) was limited to studies on so called “chronic Lyme disease” according to the initial aim. To find as many relevant studies as possible, we also used search terms as “chronic or persistent or lingering or long-term.” However, it is possible that studies that have used other descriptions for this condition may have been missed.

### Human Granulocytic Anaplasmosis

Only few studies of high quality comparing two laboratory methods have been published (Pan; Schotthoefer). One systematic review was published suggesting all three methods; microscopy of blood smear/buffy coat, PCR of blood and serology (Sanchez). However, in the acute phase of the disease, molecular detection by PCR in blood seems to have a higher sensitivity than microscopy of blood smear, and in later phase (>4 days) of disease, serology with paired samples could be preferred. In a non-systematic review (Silaghi, Table 3) it was concluded that molecular methods are preferred for direct detection of *Anaplasma* spp. in blood and tissue samples, but the sensitivity of PCR is only 68.2% in European HGA. Diagnostic methods have also been summarized in a more recent non-systematic review on eco-epidemiology and clinical management of anaplasmosis (63). Further comparative studies on evaluation of laboratory diagnostics are needed in order to be able to recommend evidence-based diagnostic methods in each phase of the disease in humans.

### Rickettsiosis

Of the various serological tests available for laboratory diagnostics of rickettsial infection, microimmunofluorescence (MIF) or IFA for detection of IgG and IgM in acute and convalescent sera are widely used (Bizzini, Kantsö) and accepted as the reference method (64). A major disadvantage includes poor sensitivity during early infection, and this is a limitation when using single sera for diagnosis. Another limitation is that the interpretation of serological data can be confused by cross-reactions with other *Rickettsia* spp. and similar to that, the species of *Rickettsia* chosen as antigen source also affects the outcome. The utility of protein immunoblots or ELISA with recombinant antigen may be an alternative (Kowalczewska, Do), but is not yet sufficiently validated (65). Molecular methods are both sensitive and specific. Real-time PCR is often used for detection, while conventional and nested PCR also have the potential for sequencing, and a number of equally useful gene targets are reported, and unique gene regions can be targeted for species identification (Boretti, Mouffok, Renvoise, Znazen). The most useful specimens, often during early infection, are swabs or skin biopsies from the “eschar” or blood (buffy coat) (66).

### Neoehrlichiosis

One high quality study with low risk of bias compared two different laboratory methods: a multiplex PCR and a singleplex real-time PCR (Quarsten). It showed a low sensitivity (6%) for the multiplex PCR and a slightly higher, but still low, sensitivity (10%) for the singleplex PCR. Plasma was found to be superior to whole blood for detection of *N. mikurensis* DNA in human samples. So far, no serologic tests have been developed for neoehrlichiosis. Further high-quality studies are needed before any recommendation for laboratory evaluation of patients with suspected neoehrlichiosis can be stated.

### Babesiosis

Golden standard for babesiosis diagnostics is still conventional blood smear. IFA serology and/or PCR can be used for confirmation of the blood smear results. Four studies (three on *Bab. microti* and one on *Bab. microti* and *Bab. divergens*) were included where serology was compared to microscopy. In all of these the serology was also compared to either PCR and/or IFA (Table 2). The sensitivity of the serological tests in three of the studies varied between 84.5 and 97.4%. In the fourth study, diagnostic accuracy could not be calculated due to the low number of samples included. Specificity varied between 97.6 and 99.5%, but was only calculated in two of the four studies. However, in all four studies the risk of bias was graded as medium to high, making it difficult to draw any firm conclusions.

Four studies compared PCR to blood smear and two studies compared PCR to blood smear and serology or conventional PCR. Most of the studies focused on *Bab. microti*, possibly because they were conducted in the US where this species is most prevalent (30). In the studies where sensitivity of the PCR assays was reported it was 100% (Table 2). In one study, the sensitivity was reported as 5–10 parasites/μl. Furthermore, two studies compared different methods of microscopy with conventional blood smear. In one of the studies (Aase), a modified microscopy protocol, called the LM method, was compared with PCR and serology. This study had a low risk of bias but no positive samples other than the positive controls, and the conclusion in the study was that the modified microscopy method was unreliable. The other study (Racsa) evaluated CellaVision, but only six samples from patients with babesiosis were included, making conclusions regarding its usefulness difficult. Taken together, there is not sufficient scientific support to change the golden standard of conventional blood smear microscopy, but PCR and IFA serology can be used as a complement when the results from the microscopy are uncertain.

According to the systematic review by Sanchez et al. microscopy on thin blood smear is the most reliable method for diagnosis of active babesiosis, evidence grading I-B (American Evidence-Based Scoring System). PCR should be considered early in the infection when parasites are few and difficult to visualize in blood smears, but should be used with caution when monitoring response to therapy since DNA can be detected for a long time after parasites are no longer visualized in blood smears (IIb-B). Serology can confirm the diagnosis (I-B), but cannot replace microscopy and PCR.

### Hard Tick Relapsing Fever

*B. miyamotoi* was discovered as a potential human pathogen as recently as 2011 (49), and so far the disease has been described in case reports and case series from Asia, Europe and North America (31, 32, 49, 50, 67, 68), and consequently, larger evaluations of diagnostic methods are lacking. The experience of clinical diagnostics originates from limited case series and case reports and have recently been summarized in a non-systematic review by Cutler et al. (69). Laboratory methods for diagnosis mainly employ PCR and serology, even though positive microscopy findings have been reported in cerebrospinal fluid (CSF) from immunocompromised patients with *B. miyamotoi*-associated meningoencephalitis (31, 32, 67). Culture in modified Kelly-Pettenkorfer medium as described by Koetsveld et al. (Table 2) is laborious, time-consuming and has a rather low sensitivity in clinical samples and is therefore mainly suited for research purposes. PCR methods targeting the 16S rRNA, glpQ or flagellin genes have been able to detect *B. miyamotoi*-specific DNA in CSF and blood samples from meningoencephalitis cases, and from blood samples from patients with systemic illness (31, 70, 71). Commercially available ELISAs based on the C6 peptide, as evaluated by Molloy et al. (Table 2), may be positive in *B. miyamotoi* disease, but are not able to distinguish between infections caused by *B. miyamotoi* and *B. burgdorferi* s.l. causing Lyme borreliosis. In contrast, glycerophosphodiester phosphodieasterase (GlpQ) antigen is present in relapsing fever *Borrelia* but not in *B. burgdorferi* s.l. and can therefore discriminate between the two (72). In a more recent study, combinations of GlpQ and Variable major proteins (Vmps) from *B. miyamotoi* increased sensitivity and/or specificity compared to single antigens (73). However, GlpQ and Vmps assays are still experimental and not yet widely available. It appears that PCR is the most suitable diagnostic method in early systemic disease, i.e., the first 1–2 (4) weeks, as the development of specific antibodies may be delayed (49). Also, development of antibody responses may be generally compromised in immunosuppressed individuals, for example patients treated with rituximab (31, 32, 50, 67). However, establishment of more precise recommendations for laboratory testing in suspected *B. miyamotoi* disease will need further evaluation.

### Tularemia

Tick bites are the most common mode of transmission for *F. tularensis* subsp. *tularensis* to humans in the USA (74). The presence of the less virulent *F. tularensis* subsp. *holarctica* in European ticks has been described (19–21), but transmission of tularemia via ticks is relatively uncommon (53, 75, 76). Laboratory confirmation of tularemia consists of detecting the bacteria in a biological sample and/or detecting a specific antibody response. The seven articles included in this review were on serological methods, including one in conjunction with PCR. All studies were assessed as having a medium risk of bias regarding clinical materials. The performance of serology is adequate for diagnosis in cases with a typical presentation (ulceroglandular tularemia), with caution for serological cross-reactions. The varying specificity of serological tests should, whenever possible, prompt confirmation with PCR of biological material in atypical presentations, especially in a low-prevalence setting.

### Bartonellosis

In *I. ricinus* ticks, *Bartonella* spp. are found variably in 0–30% (18, 77–79). However, tick-borne transmission of *Bartonella* spp. to humans has not definitely been established, despite the detection of specific antibodies in 15–33% of individuals with LB (80, 81).

Among the 25 articles evaluating diagnostic methods, there was none assessed as being of high quality. Most of them were non-clinical laboratory comparisons of methods, either serological or PCR. The recommendation from the European Centre for Disease Prevention and Control regarding diagnostics in suspected bartonellosis consists of bacterial culture, PCR and serology in combination, but has not been consequently applied.

### Tick-Borne Co-infections

The lack of eligible articles focusing on human tick-borne co-infections highlights the need for further studies.

### Persisting Post-treatment Lyme Borreliosis; “Chronic Lyme Borreliosis”

The terms post-treatment Lyme borreliosis/disease, chronic Lyme borreliosis/disease and persisting post-treatment Lyme borreliosis/disease are interchangeably used in the scientific contexts to describe a heterogenous patient population with mainly unspecific symptoms, either attributable to LB or not, following recommended antibiotic treatment of LB (82–85). In this systematic literature search, we included several search terms usually used to describe the phenomenon, to cover the whole scientific spectrum of published papers. Following the first broad search, 16 review articles and four articles comparing one or more methods were assessed for eligibility. However, none of the papers fulfilled the inclusion criteria. We conclude that to date, science has no alternative diagnostic tests to offer patients with persisting symptoms post-treatment besides the well-established ones recommended for investigation of LB. In a recently published report, however, it has been shown that symptoms that are often categorized as chronic LB in the general debate could not be uniquely linked to LB (86). Instead, ~20% of the total group of patients showed signs of autoimmunity. Further studies are needed to confirm these results, but the findings may provide an alternative explanation for this medical controversy and indicate that diagnostic tests for these conditions need a different focus.

## Conclusions

Taken together, the number of published studies and systematic reviews regarding the accuracy of diagnostic tests for TBDs, other than LB and TBE, evaluated on clinical samples, were unexpectedly limited. Many of the studies have been performed on a small number of study participants using a case control study design. When assessing these studies according to the QUADAS checklist, many of them were classified as having a medium to high risk of bias. This is of course a highly relevant problem when evaluating patients with complaints possibly related to tick bite(s). Which microbes should be tested for and what laboratory methods should be used? Unfortunately, our systematic review reveals that high quality clinical evaluations of which laboratory methods to use for diagnosis of most of the listed TBDs are scarce. However, one should also realize that cross sectional studies, that are often considered to be of higher quality than case control studies, are difficult to perform on infectious diseases that occur with low frequency in the population. Consequently, we need to accept case control studies together with epidemiological studies and case series. Admittedly, one needs to keep in mind that a medium to high risk of bias according to the QUADAS checklist does not necessarily imply poor quality of the study with regard to evaluation of test performance, since major factors of importance are inclusion of well-defined clinical cases and relevant controls.

For diagnosis of TBDs other than LB and TBE, a number of different laboratory techniques have been used, such as blood smear microscopy, immunohistochemistry, culture, serology and PCR. Which method that is most suitable partly depends on during which phase of the disease the samples are taken. Two or three methods are preferably combined in order to achieve higher sensitivity. For most of the TBDs covered in this systematic review, only few studies fulfilled the inclusion criteria for in-depth evaluation, and several of them were based on small study populations. There were no eligible evaluation studies for tick-borne co-infections or for persistent LB after antibiotic treatment. Our findings highlight the need for larger evaluations of laboratory tests using clinical samples from well-defined cases taken at different time-points during the course of the diseases. Since the TBDs occur with low frequency in the population, single-center cross-sectional studies are practically not possible, but multi-center case control studies using well-defined clinical cases and relevant controls could be a way forward.

## Data Availability Statement

The original contributions presented in the study are included in the article, further inquiries can be directed to the corresponding author.

## Author Contributions

All authors listed have made a substantial, direct and intellectual contribution to the work, and approved it for publication.

## Funding

This work was supported by the Norwegian Directorate of Health and by grants of the European Union through the European Development fund and the Interreg Öresund-Kattegat-Skagerrak and the Interreg NorthSea Region Programmes 2014-2020 as part of the ScandTick Innovation project (reference number 2015-29 000167) and the NorthTick project (reference number J-No: 38-2-7-19).

## Conflict of Interest

The authors declare that the research was conducted in the absence of any commercial or financial relationships that could be construed as a potential conflict of interest.

## Publisher's Note

All claims expressed in this article are solely those of the authors and do not necessarily represent those of their affiliated organizations, or those of the publisher, the editors and the reviewers. Any product that may be evaluated in this article, or claim that may be made by its manufacturer, is not guaranteed or endorsed by the publisher.
